# CORUM: the comprehensive resource of mammalian protein complexes—2019

**DOI:** 10.1093/nar/gky973

**Published:** 2018-10-24

**Authors:** Madalina Giurgiu, Julian Reinhard, Barbara Brauner, Irmtraud Dunger-Kaltenbach, Gisela Fobo, Goar Frishman, Corinna Montrone, Andreas Ruepp

**Affiliations:** Institute for Bioinformatics and Systems Biology (IBIS), Helmholtz Zentrum München—German Research Center for Environmental Health (GmbH), Ingolstädter Landstraße 1, D-85764 Neuherberg, Germany

## Abstract

CORUM is a database that provides a manually curated repository of experimentally characterized protein complexes from mammalian organisms, mainly human (67%), mouse (15%) and rat (10%). Given the vital functions of these macromolecular machines, their identification and functional characterization is foundational to our understanding of normal and disease biology. The new CORUM 3.0 release encompasses 4274 protein complexes offering the largest and most comprehensive publicly available dataset of mammalian protein complexes. The CORUM dataset is built from 4473 different genes, representing 22% of the protein coding genes in humans. Protein complexes are described by a protein complex name, subunit composition, cellular functions as well as the literature references. Information about stoichiometry of subunits depends on availability of experimental data. Recent developments include a graphical tool displaying known interactions between subunits. This allows the prediction of structural interconnections within protein complexes of unknown structure. In addition, we present a set of 58 protein complexes with alternatively spliced subunits. Those were found to affect cellular functions such as regulation of apoptotic activity, protein complex assembly or define cellular localization. CORUM is freely accessible at http://mips.helmholtz-muenchen.de/corum/.

## INTRODUCTION

Understanding biological processes at cellular and system levels is an important task in all living organisms. Protein complexes play critical roles in an array of biological processes, including protein synthesis, signaling and cellular degradation processes. To date, there are no reliable estimates about the total number of protein complexes in cells (complexome), but data from single cell organisms provide evidence that more than half of the gene products are involved in the formation of protein complexes (1). According to estimates from Berggård *et al.* (2), even more than 80% of proteins work in complexes. Many proteins are subunits of more than one complex, which extends the number of potential protein complexes. The RING-box protein 1 (RBX1), that was present in 35 protein complexes in the 2009 release of CORUM (3), is now found in 65 protein complexes.

Due to the importance of the topic, several endeavors were undertaken in order to unravel the cellular complexome. The first large-scale screens for protein complexes were performed in budding yeast (4,5) and discovered 491 and 547 complexes, respectively. Recent analyses of the interactome/complexome in human cells revealed a wealth of novel information (6–8). Integration of published datasets in the human protein complex map (hu.MAP) resulted in 4659 complexes composed from 7777 unique proteins (9).

For many years, the composition of thousands of protein complexes has been analysed in individual experiments and published in the scientific literature. In addition to the identification of subunit composition, which is the standard in high-throughput experiments, these complexes are also characterized with respect to their cellular function, association with diseases and sometimes stoichiometry. In order to provide a high-quality resource of information on mammalian protein complexes, we generated the comprehensive resource of mammalian protein complexes (CORUM) with 1750 complexes in the first release (10). The CORUM release 3.0 presents a significantly extended dataset that now consists of 4274 mammalian protein complexes. A graphical analysis tool was implemented that displays potential protein–protein interactions between the subunits of protein complexes. The tool is based on the Cytoscape javascript library and uses data of mammalian organisms from the IntAct database. At last, we present a collection of 58 protein complexes, where alternative splice variants result in altered complex function or affect diseases. CORUM is freely accessible at http://mips.helmholtz-muenchen.de/corum/.

## DATABASE DESCRIPTION AND NEW DEVELOPMENTS

### Design and application of CORUM

Our goal was the generation of a reference dataset of protein complex information from mammalian organisms. In order to obtain a high quality and reliability of the data, we only include protein complexes that have been isolated and characterized in individual experiments. Although protein complex information from high-throughput approaches provides an invaluable resource of novel information, we do not include it in CORUM as these data are usually not corroborated by experiments unraveling the biological function of complexes. As of note, our novel core set from CORUM 3.0 (3512 protein complexes) and the hu.MAP dataset share only 29 identical protein complexes (9). Experienced biocurators critically extract information from the scientific literature and transfer it into CORUM using established vocabularies and stable identifiers from well-known resources such as UniProt and Gene Ontology. The major focus for the application of CORUM lies on network biology and network medicine. Hence, the dataset is biased toward protein complexes which consist of at least two different subunits. According to Teichmann (11), the majority of (known) protein complexes in PDB are homomers. Based on recent statistics from PDB, 9206 homomers and 2677 heteromeric protein structures have been determined using X-ray crystallography. Of note, these data cover all groups of organisms. In the UniProt release 06_2018, 2344 proteins from the taxon Homo sapiens were annotated either as homodimer or homotetramer (12). In contrast, CORUM includes only 126 homomers. Apart from this exception, CORUM aims to offer a representation of the complexome of mammalian cells.

**Figure 1.**
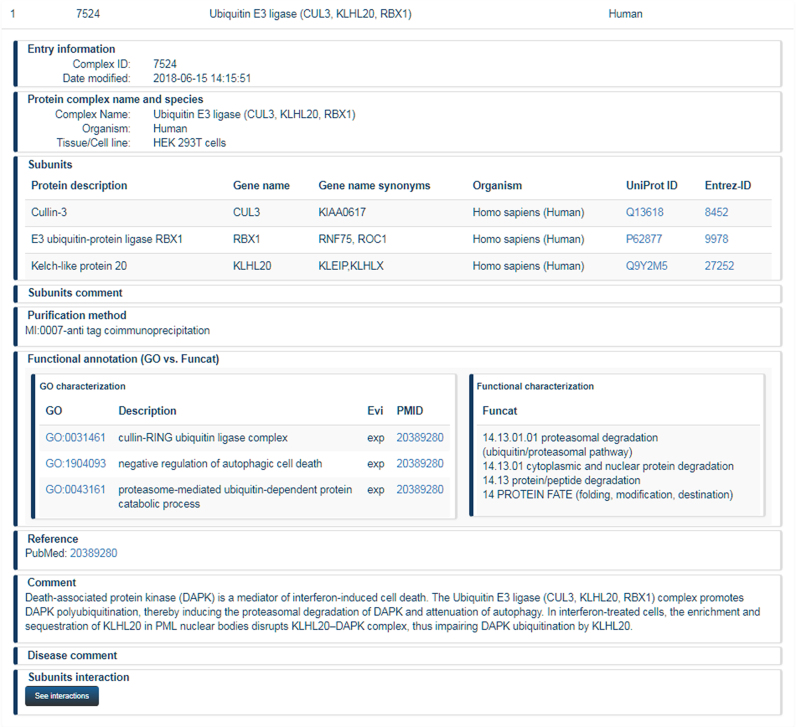
Annotation of protein complexes in CORUM. Presentation of the Ubiquitin E3 ligase (CUL3, KLHL20, RBX1) complex in CORUM. Information about functional annotation using term from Gene Ontology is automatically translated into respective terms from the FunCat annotation scheme.

Since the previous CORUM release, basic annotation topics such as protein complex name, identification of subunits based on UniProt identifiers, references of used articles and comments remained unchanged. For functional annotation of protein complexes, we offer a mapping to Gene Ontology (GO) terms since CORUM 2.0. In the meantime, we switched manual functional annotation of novel complexes completely to GO terms (Figure 1).

**Figure 2.**
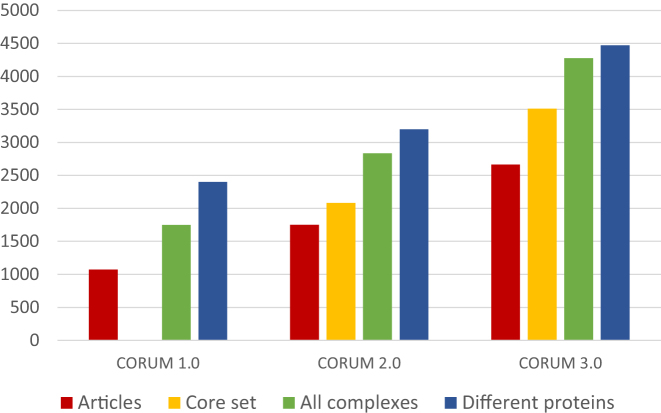
Data growth in CORUM. The plot compares the data content of CORUM versions 1.0, 2.0 and 3.0. It includes the number of articles that were used to create the datasets, the number of core set complexes, the total number of protein complexes as well as the total number of different proteins that are found in the dataset. As we did not provide a core set in CORUM 1.0, a respective number is missing.

Compared to the previous CORUM publication (3), the content of the dataset has increased from 2837 to 4274 protein complexes (Figure 2). In particular, the core set has grown considerably. The core set is a subset of CORUM that is reduced by eliminating redundant information such as protein complexes that were characterized from different mammalian species or complexes which were isolated by different methods. The core set now includes 3512 protein complexes, which is a gain of 70% compared to CORUM 2.0. Regarding the organisms that were used to characterize protein complexes, there are only minor changes comparing to the previous CORUM publication. The largest fraction of complexes was isolated from Homo sapiens (67%) followed by mouse (15%) and rat (10%). Other complexes were isolated from organisms such as cattle, pig, rabbit or are composed of subunits from different organisms. The growth of the CORUM dataset is also accompanied by a higher total number of different gene products that are used as protein complex subunits. While in CORUM 2.0 complexes were composed of 3189 different proteins, the number now increased to 4473. This represents 22% of all known protein-coding genes according to the Ensembl database version 92.38 (13).

In recent years, the CORUM dataset was used in a large number of analyses as reference dataset, for benchmarking computational models and high-throughput experimental data. Examples are results from three pioneering high-throughput investigations that were combined in order to present a comprehensive complexome of human cells (9). In addition, protein complex information from CORUM is increasingly applied for the analyses of disease mechanisms. Use cases are network-based in silico drug efficacy screening (14), pituitary hormone deficiency in inherited gingival fibromatosis (15) or the architecture of human protein communities and disease networks (16). In the area of diseases, the CORUM dataset is particularly often applied in cancer analyses, demonstrated by more than ten citations during the past 2 years. These include protein-interaction network associated analyses of MLL(KMT2A)-fusion proteins in leukemia (17) and the detection of dysregulated protein-association networks in breast cancer cell lines (18).

Beside the analysis of experimental data, CORUM is also used by other data resources such as ‘Interactome INSIDER’ (19) or the ‘MouseNet v2’ database of gene networks (20). At last, CORUM is cross-referenced by the UCSC-Genome browser (21) and UniProt (12).

### Annotation of splice variant complexes and their functional implications

To generate a high number of different gene products from comparably small genomes, mammals make use of processes such as alternative splicing to produce more than 82 335 distinct mRNAs e.g. in humans, according to the Gencode release 28 (22). Accordingly, mammalian protein complexes also exist in different isoforms, with the composition varying across cell types and conditions (23). In our new CORUM release, we provide a dataset of 58 protein complexes, which contain alternatively spliced subunits. Those subunits were found to alter cellular functions such as methylation of nucleosomal histone H3-lysine 27 by the PCM3 complex (with EED isoform 3) whereas the PRC2 complex (with EED isoform 1) preferentially methylates nucleosomal histone H1-lysine 26 (24). Examples, where alternative splicing affects protein complex functions via altered protein binding are the caspase-2 gene products. The short isoform, CASP-2S, inhibits apoptosis, whereas the long isoform, CASP-2L, promotes apoptosis. The CASP-2S–fodrin complex inhibits DNA damage-induced cytoplasmic fodrin cleavage. This process inhibits membrane blebbing and phosphatidylserine externalization that are indicative of apoptosis in cancer cells. The molecular basis for the different activities of the two CASP variants is that, in contrast to CASP-2S, the long isoform CASP-2L does not interact with fodrin (25).

The effect of alternatively spliced subunits of cytoskeletal protein dystrophin can be illustrated for DAPCs (dystrophin-associated protein complexes). Duchenne muscular dystrophy is caused by the absence of dystrophin (26). The DAPC is destabilized when dystrophin is absent, which leads to downregulated levels of the member proteins (27). Two dystrophin 71 isoforms (Dp71d and Dp71f) form multi-protein complexes in the hippocampal neurons. Dp71d-DAPC is mainly localized in bipolar GABAergic and Dp71f–DAPC in multipolar glutamatergic hippocampal neurons. The subunit composition of these protein complexes seems to affect their neuronal phenotype (28). It has been described that Dp71d–DAP complexes were present only in the nuclei of non-neuronal cells (29). However, recently it was demonstrated for the first time that isoform-containing complexes Dp71f–DAP and Dp71fd–DAP were localized in the nucleus of primary hippocampal neurons (28). This example shows that alternative splicing variants as subunits may regulate not only specific activities but also the tissue-specific localization of protein complexes.

The splice variant dataset illustrates that functional properties of particular protein complexes can only be represented by isoform-specific variant annotation. The splice variant complexes can be downloaded as a separate dataset.

### Prediction of protein–protein interactions within protein complexes

Although there is a growing number of protein complexes with structure information, characterization of complexes is usually restricted to the identification of the subunits and biomedical information such as cellular function or association with diseases. Structural information such as stoichiometry of individual subunits is rarely discovered. In articles used for CORUM, we found only 288 protein complexes with stoichiometry data. For detailed structural information discovered by x-ray crystallography or nuclear magnetic resonance, information is even more sparse. In the new CORUM dataset 3.0, we have annotated 109 protein complexes with the PSI–MI interaction method, MI:0114 x-ray crystallography’. Preliminary information about protein complex structure with respect to neighboring proteins can be performed by serial protein interaction experiments. This was successfully applied for elucidation of the chaperonin Tric/CCT (30).

In order to provide users with the option to inspect putative interactions between complex subunits, the new CORUM release provides a graphical tool that integrates known PPI information. A large publicly available collection of protein–protein interactions is provided by the IntAct database which includes data from different resources (31). For the CORUM tool, we used the ‘physical association’-interactions of IntAct, release 05 November 2018, from the organisms *Homo sapiens, Rattus norvegicus* and *Mus musculus*. The largest fraction of PPI information was obtained from Homo sapiens (238 841 entries), followed by mouse (15 138) and rat (3962).

**Figure 3.**
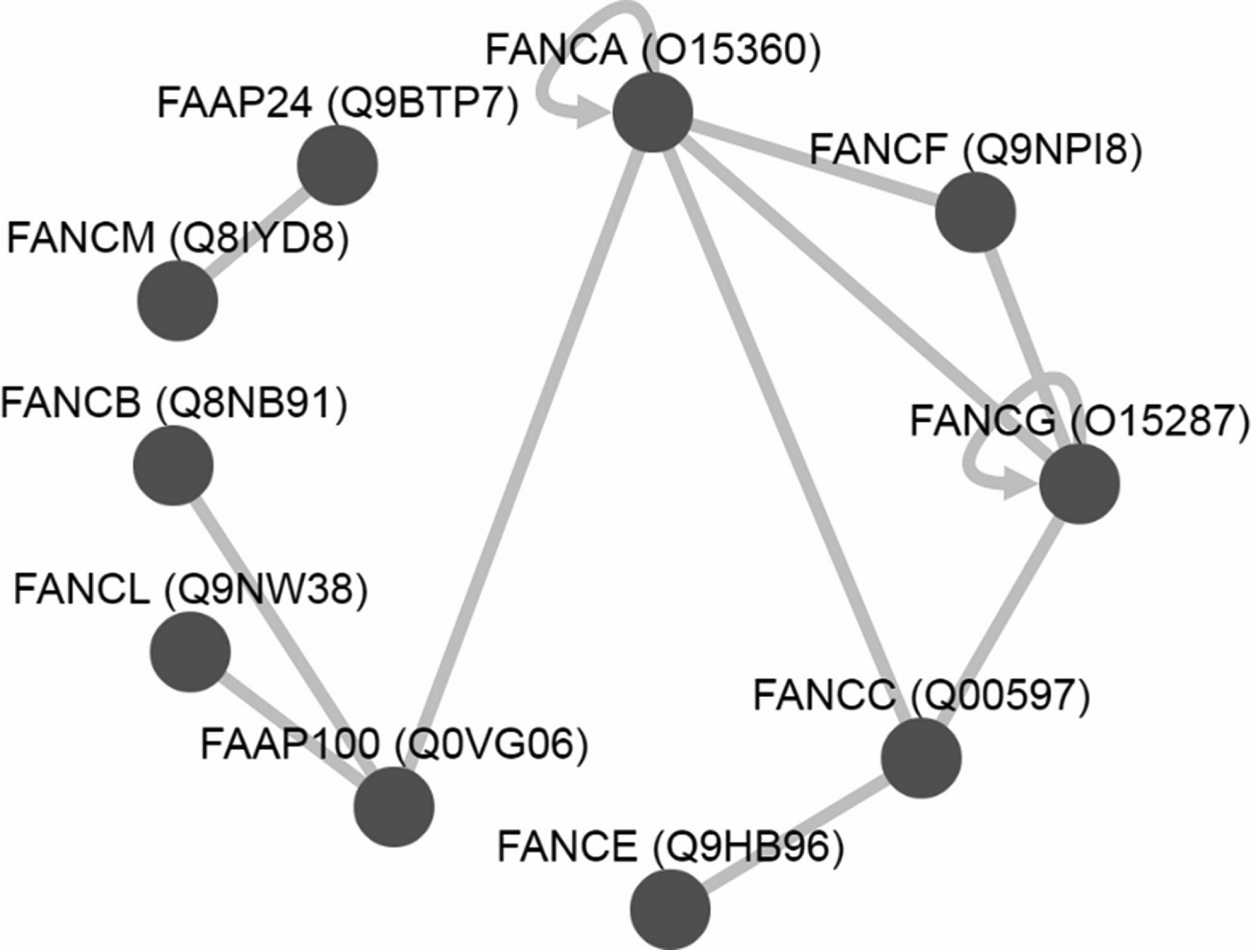
Protein–protein interactions between protein complex subunits. Based on data from the IntAct database, validated protein–protein interactions of the Fanconi anemia FAAP100 complex (complex 6884) are displayed with Cytoscape.

For visualization of interactions between protein complex subunits we use Cytoscape. Cytoscape.js is a javascript-based graph-visualization library that we embedded in version 3.2 on our website. For each protein complex that contains at least one interaction we show all protein–protein interactions according to the IntAct dataset in a graph below the list of search results. For Fanconi anemia FAAP100 complex (complex 6884) for example, nine interactions between subunits and two self-associations were found (Figure 3). Five interactions between FANCA, FANCC, FANCF and FANCG give rise to the speculation that these four proteins build a core of the complex. This is corroborated by analyses of four forms of the Fanconi anaemia core complexes from different subcellular compartments (32). All forms of the complex contained at least these four proteins.

The fact, that a protein–protein interaction was discovered in an experiment is no proof that it also exists in a protein complex. However, it is tempting to assume that for the majority of protein complexes, PPI data provide a reliable prediction of intramolecular associations.

## CONCLUSION

CORUM is a publicly available, centralized database for mammalian protein complexes based on manually curated information from scientific literature. Basic and translational researchers are provided with extensive search options to look for complexes containing their genes of interest, exhibiting a specific biological function or displaying other features. Computational biologists can download entire datasets in different formats for advanced studies. The importance of protein complex data is demonstrated by hundreds of studies that used the CORUM dataset during the last decade. These include basic research such as the inventory of the mammalian complexome as well as applied biomedical research, in particular of cancer. The CORUM 3.0 release provides a substantially enlarged dataset of mammalian complexes which is accompanied by a wider coverage of gene products that serve as subunits of protein complexes.

In recent years, a growing number of studies has demonstrated that variants of gene products may have substantial effects on protein complex function. Here, we present for the first time a dataset that covers the impact of splice variants on cellular processes and diseases.

In addition to the larger dataset, the CORUM 3.0 release also presents a Cytoscape-based tool for the graphical representation of known protein–protein interactions of subunits. As structural information about large protein complexes is sparse, this approach allows the prediction of structurally adjacent proteins within these large complexes.

An important goal of CORUM for the future will be to obtain an even more complete representation of experimentally characterized protein complexes. To achieve this goal, we welcome the input of other researchers sending us information about novel published protein complexes not yet included in the dataset. Please contact us at andreas.ruepp@helmholtz-muenchen.de.
